# Non-Canonical Wnt Signaling Regulates Cochlear Outgrowth and Planar Cell Polarity via Gsk3β Inhibition

**DOI:** 10.3389/fcell.2021.649830

**Published:** 2021-04-16

**Authors:** Andre Landin Malt, Shaylyn Clancy, Diane Hwang, Alice Liu, Connor Smith, Margaret Smith, Maya Hatley, Christopher Clemens, Xiaowei Lu

**Affiliations:** Department of Cell Biology, University of Virginia Health System, Charlottesville, VA, United States

**Keywords:** non-canonical Wnt, Gsk3β, Rac1, planar cell polarity, hair bundle, kinocilium, cochlea

## Abstract

During development, sensory hair cells (HCs) in the cochlea assemble a stereociliary hair bundle on their apical surface with planar polarized structure and orientation. We have recently identified a non-canonical, Wnt/G-protein/PI3K signaling pathway that promotes cochlear outgrowth and coordinates planar polarization of the HC apical cytoskeleton and alignment of HC orientation across the cochlear epithelium. Here, we determined the involvement of the kinase Gsk3β and the small GTPase Rac1 in non-canonical Wnt signaling and its regulation of the planar cell polarity (PCP) pathway in the cochlea. We provided the first *in vivo* evidence for Wnt regulation of Gsk3β activity via inhibitory Ser9 phosphorylation. Furthermore, we carried out genetic rescue experiments of cochlear defects caused by blocking Wnt secretion. We showed that cochlear outgrowth was partially rescued by genetic ablation of Gsk3β but not by expression of stabilized β-catenin; while PCP defects, including hair bundle polarity and junctional localization of the core PCP proteins Fzd6 and Dvl2, were partially rescued by either Gsk3β ablation or constitutive activation of Rac1. Our results identify Gsk3β and likely Rac1 as downstream components of non-canonical Wnt signaling and mediators of cochlear outgrowth, HC planar polarity, and localization of a subset of core PCP proteins in the cochlea.

## Introduction

Wnt signaling regulates a plethora of developmental processes through the canonical β-catenin-dependent pathway and the non-canonical β-catenin-independent pathway (Komiya and Habas, 2008; Wiese et al., 2018). Upon Wnt ligand binding to the Frizzled receptor, non-canonical Wnt signaling controls cell polarity and morphogenetic movements through the Rho family small GTPases or heterotrimeric G-proteins. In addition, the evolutionarily conserved planar cell polarity (PCP) pathway is a key regulator of tissue morphogenesis, whereby asymmetric localized core PCP protein complexes orient cell polarity and drive polarized cell behaviors within the plane of the tissue (Devenport, 2014). Specifically, two opposing asymmetric protein complexes, one consisting of homologs of Frizzled (Fzd) and Dishevelled (Dvl), and the other Van Gogh and Prickle, bridged across cell membranes by Flamingo (homolog of Celsr1-3), generate a polarity vector across the tissue plane (Butler and Wallingford, 2017). Because the non-canonical Wnt and PCP pathways share many components, including the Fzd receptor, as well as the effectors Dvl and Rho GTPases, the PCP pathway is often considered to be a branch of non-canonical Wnt signaling. However, emerging evidence suggests divergence of, and crosstalk between, the mammalian non-canonical Wnt and PCP pathways. The mammalian genome encodes 19 Wnt, 10 Fzd, and 3 Dvl genes. Fzd3/6 are components of the mammalian PCP pathway (Wang et al., 2006; Chang et al., 2016); however, to date, their specific Wnt ligands have not been identified (Sato et al., 2010; Yu et al., 2012; Voloshanenko et al., 2017). On the other hand, we and others have recently demonstrated that secreted Wnts are required for asymmetric localizations of a subset of PCP proteins in inner ear sensory epithelia, including Fzd3/6 and Dvl2 (Landin Malt et al., 2020; Najarro et al., 2020). Importantly, we have shown that asymmetric localization of Fzd6 is controlled by a Wnt/G-protein/PI3K signaling pathway (Landin Malt et al., 2020). In this study, we leverage the inner ear sensory epithelium and genetic tools available to further illuminate the precise relationship between the mammalian non-canonical Wnt and PCP pathways.

The mouse cochlear sensory epithelium, or the organ of Corti (OC), is a well-established system for studying PCP signaling (Tarchini and Lu, 2019). Crucial for their function as sound receptors, hair cells (HCs) in the OC project on their apical surface a V-shaped hair bundle consisting of rows of actin-based stereocilia organized in a staircase pattern. The vertices of all hair bundles are uniformly aligned along the medial-lateral axis of the cochlear duct. The polarized structure of hair bundles and other apical cytoskeletal elements define cell-intrinsic PCP (iPCP), while uniform hair bundle orientation is a hallmark of tissue-level PCP. Hair bundle formation is coincident with the migration of the microtubule-based kinocilium, which migrates to, and anchors at, the lateral edge of the HC and is tethered to the nascent hair bundle at its vertex. Thus, kinocilium positioning is crucial for hair bundle polarity and orientation. This process is coordinately controlled by intercellular PCP signaling, several iPCP signaling modules, and a novel, non-canonical Wnt/G-protein/PI3K signaling pathway (Tarchini and Lu, 2019; Landin Malt et al., 2020). To shed light on the crosstalk and integration of these signaling pathways, we sought to identify cochlear effectors of non-canonical Wnt signaling. Specifically, we focused on two candidates: the small GTPase Rac1 and the kinase Gsk3β. Rac1 has been shown to be activated by non-canonical Wnt signaling in cultured cells and mediate one of the iPCP signaling modules in the OC (Grimsley-Myers et al., 2009; Sato et al., 2010; Landin Malt et al., 2019). On the other hand, Gsk3β activity is inhibited by both canonical Wnt and PI3K/Akt signaling (Metcalfe and Bienz, 2011; Beurel et al., 2015). Here, we report that epithelium-secreted Wnts promote inhibitory phosphorylation of Gsk3β at Ser9 (S9) in the OC *in vivo*. We further show that cochlear growth, hair bundle polarity, and core PCP protein localization defects caused by blocking Wnt secretion are partially rescued by genetic ablation of Gsk3β in the cochlear epithelium, and to a lesser extent, constitutive activation of Rac1. Together, these findings identify both Gsk3β and Rac1 as effectors of non-canonical Wnt signaling crucial for hair bundle morphogenesis and cross-regulation of the PCP pathway.

## Results

### Wnt Signaling Regulates Gsk3β Activity via Serine 9 Phosphorylation in the Cochlea

**FIGURE 1:**
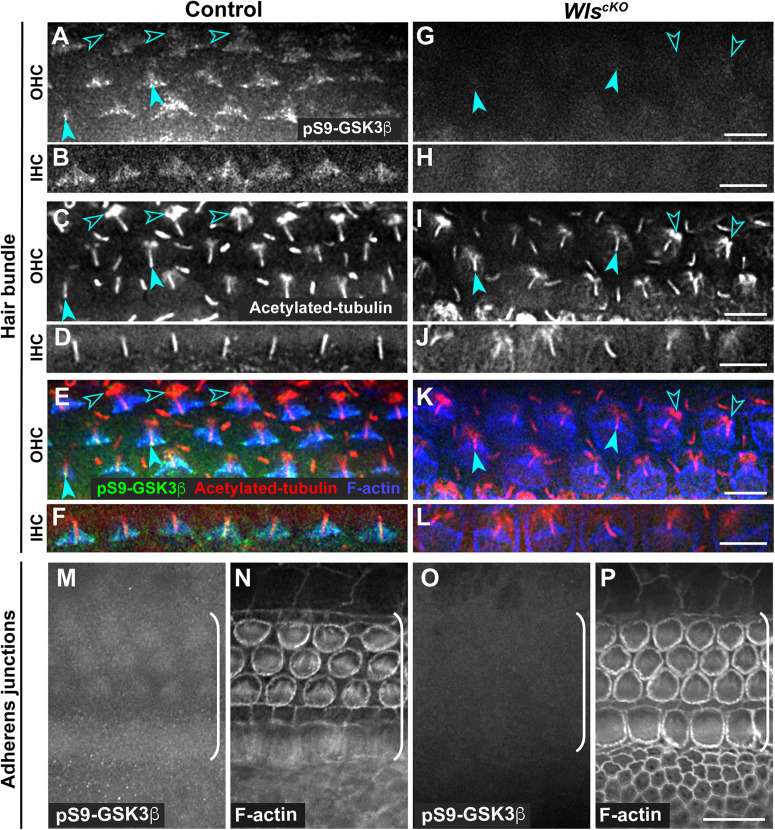
Wnts promote inhibitory phosphorylation of Gsk3β at Ser9 in the cochlea. **(A–L)** pS9-Gsk3β (green), acetylated-tubulin (red), and phalloidin (blue) staining at the level of the hair bundle in control **(A–F)** and *Wls*^*cKO*^ **(G–L)** OC at E18.5. Open arrowheads indicate the pericentriolar region. Arrowheads indicate the tip of the kinocilium. **(M–P)** pS9-Gsk3β and phalloidin staining at the level of adherens junctions in control **(M,N)** and *Wls*^*cKO*^ **(O,P)** OC and surrounding regions. Brackets indicate the OC. Lateral is up. Scale bars: **(A–L)**, 6 μm; **(M–P)**, 10 μm.

**FIGURE 2:**
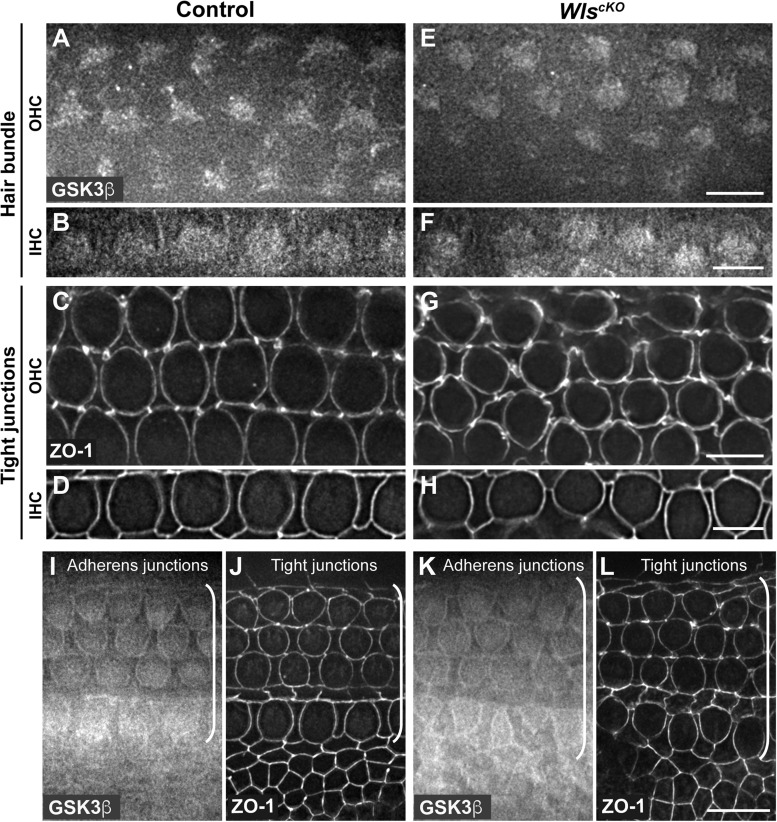
Similar Gsk3β localizations in control and *Wls*^*cKO*^ cochleae. **(A–H)** Gsk3β staining at the level of hair bundle and ZO-1 staining at the level of tight junctions in control **(A–D)** and *Wls*^*cKO*^ **(E–H)** OC at E18.5. **(I–L)** Gsk3β staining at the level of adherens junctions and ZO-1 staining at the level of tight junctions in control **(I,J)** and *Wls*^*cKO*^ **(K,L)** OC and surrounding regions. Brackets indicate the OC. Lateral is up. Scale bars: **(A–L)**, 6 μm; **(M–P)**, 10 μm.

Conditional deletion of *Wntless* (*Wls*) driven by *Emx2*^*Cre*^ (*Wls*^*cKO*^) blocked Wnt secretion from the cochlear epithelium, resulting in stunted cochlear outgrowth and both PCP and iPCP defects. We have identified PI3K as a key effector of non-canonical Wnt signaling in the OC; PI3K activity was decreased in the *Wls*^*cKO*^ OC, and importantly, PI3K activation rescued most of the *Wls*^*cKO*^ cochlear phenotypes (Landin Malt et al., 2020). However, the downstream targets of PI3K crucial for cochlear morphogenesis remain unknown. Because PI3K activation of Akt leads to inhibitory phosphorylation of Gsk3β at S9 (Beurel et al., 2015), we examined the localization of pS9-Gsk3β as well as total Gsk3β in *Wls*^*cKO*^ cochleae, using commercial knockout (KO)-validated anti-pS9-Gsk3β and anti-Gsk3β antibodies. In the control cochlea at embryonic day (E)18.5, pS9-Gsk3β was enriched in the pericentriolar region (Figures 1A–F, open arrowheads), the tip of the kinocilium (Figures 1A–F, arrowheads), and the hair bundle in both inner and outer hair cells (IHCs and OHCs) (Figures 1A–F). In addition, diffused cytoplasmic staining of pS9-Gsk3β was detected in HCs, neighboring supporting cells (SCs), and non-sensory cells surrounding the OC (Figures 1M,N). In contrast, pS9-Gsk3β staining was greatly diminished at all subcellular locations in *Wls*^*cKO*^ cochleae (Figures 1G–L,O,P). On the other hand, total Gsk3β localization in *Wls*^*cKO*^ cochleae was similar to the control; Gsk3β was localized to the hair bundle (Figures 2A–H), at the adherens junctions in the OC and in the cytoplasm of both sensory and non-sensory cells (Figures 2I–L). The specificity of the observed staining patterns of pS9-Gsk3β and total Gsk3β was confirmed by their absence in the *Gsk3*β^*cKO*^ cochleae driven by *Emx2*^*Cre*^ (Supplementary Figures 1, 2). Thus, we conclude that epithelium-secreted Wnts regulate Gsk3β activity by promoting S9 phosphorylation in the developing cochlea.

### Wnts Regulate Cochlear Outgrowth in Part Through Gsk3β Inhibition

**FIGURE 3:**
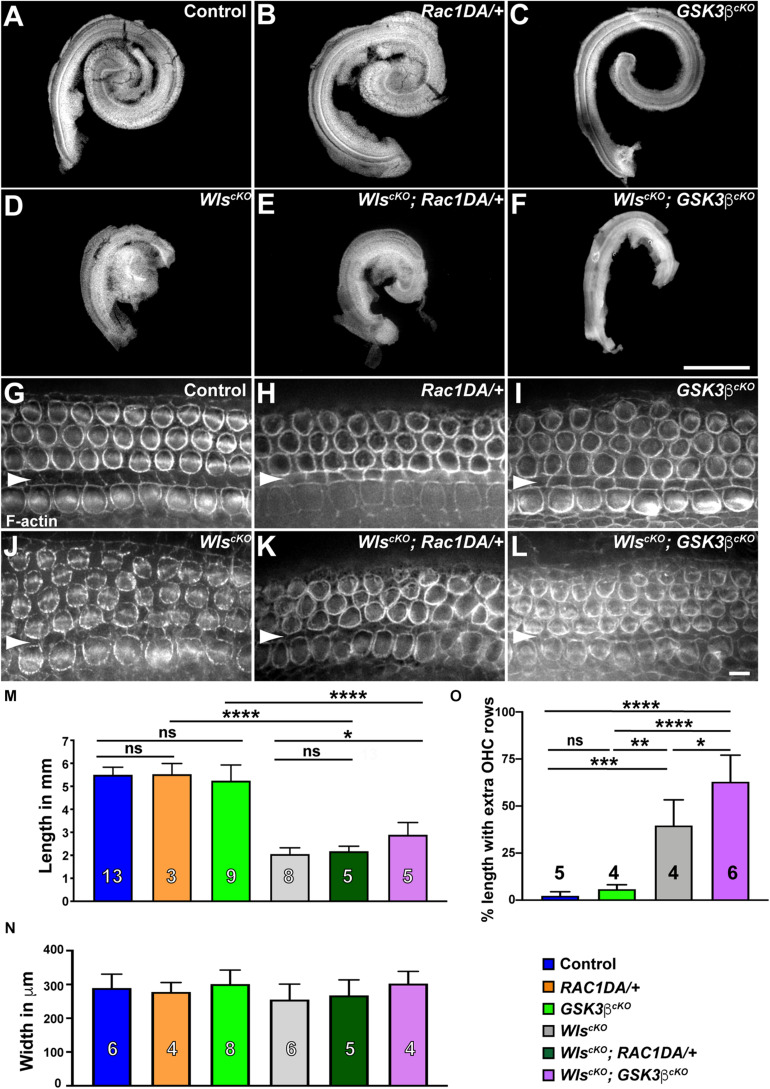
Gsk3β inactivation but not Rac1 activation partially rescued cochlear outgrowth of *Wls*^*cKO*^ mutant. **(A–F)** Dissected cochlear ducts from E18.5 wild-type control (**A**), *Rac1DA/*+ **(B)**, *Gsk3*β^*cKO*^ **(C)**, *Wls*^*cKO*^ **(D)**, *Wls*^*cKO*^; *Rac1DA/*+ **(E)**, and *Wls*^*cKO*^; *Gsk3*β^*cKO*^ **(F)** mutants. **(G–L)** Flat-mounted OC from the middle region (40–60% cochlear length) of wild-type control **(G)**, *Rac1DA/*+ **(H)**, *Gsk3*β^*cKO*^ **(I)**, *Wls*^*cKO*^ **(J)**, *Wls*^*cKO*^; *Rac1DA/*+ **(K)**, and *Wls*^*cKO*^; *Gsk3*β^*cKO*^ **(L)** cochleae stained by phalloidin. Arrowheads indicate the inner pillar cell row. Lateral is up. Scale bars: **(A–F)**, 1 mm; **(G–L)**, 6 μm. **(M–O)** Quantifications of cochlear length **(M)**, cochlear duct width **(N)**, and presence of extra OHC rows **(O)** in genotypes indicated by the color keys. Cochlear duct width **(N)** was not significantly different in all pair-wise comparisons. The number of cochleae analyzed is indicated. Ns, not significant.

Activation of Rac1 by non-canonical Wnt signaling has been well established in cultured cells; therefore, we hypothesized that Rac1 mediates non-canonical Wnt signaling in the cochlea. To test this, we asked whether constitutive activation of Rac1 was able to rescue cochlear defects of *Wls*^*cKO*^ mutants. Specifically, we crossed a Cre-inducible, constitutively active Rac1-G12V transgene (*R26-LSL-Rac1DA*) into *Wls*^*cKO*^ embryos. We first measured the length of *Wls^cKO^*; *Rac1DA/*+ compound mutant cochleae at E18.5. As a control, expression of Rac1-G12V in the cochlear epithelium driven by *Emx2*^*Cre*^ (*Rac1DA/*+) did not significantly alter cochlear length, width, or OC patterning (Figures 3A,B,G,H,M,N). Moreover, Rac1-G12V expression in *Wls*-deficient cochlear epithelia did not rescue the cochlear length (Figures 3D,E,J,K,M).

Next, we analyzed the *Gsk3*β^*cKO*^ cochleae to determine the role of Gsk3β in Wnt-mediated cochlear outgrowth. At E18.5, *Gsk3*β^*cKO*^ cochleae had largely normal length and OC patterning (Figures 3A,C,G,I,M–O), suggesting that the function of Gsk3 in cell fate regulation at earlier stages was spared in *Gsk3*β^*cKO*^ mutants (Ellis et al., 2019). In contrast to Rac1 activation, the length of the *Wls^cKO^*; *Gsk3*β^*cKO*^ compound mutant cochleae was partially but significantly rescued, and an intermittent extra OHC row was present along ∼60% of the total cochlear length (Figures 3D,F,J,L–O). As Gsk3β inactivation, but not Rac1 activation, partially rescued cochlear outgrowth defects of *Wls*^*cKO*^ mutants, we conclude that Wnt signaling promotes cochlear outgrowth in part through Gsk3β inhibition.

### Expression of Stabilized β-Catenin Failed to Rescue Outgrowth Defects of *Wls*^*cKO*^ Cochleae

Gsk3 inhibition is a key step in canonical Wnt signaling; sequestration of Gsk3 prevents phosphorylation of β-catenin, thereby stabilizing β-catenin, which translocates into the nucleus and partners with TCF transcription factors to activate Wnt target gene expression (Wiese et al., 2018). During cochlear development, canonical Wnt signaling promotes cell proliferation of otic precursors in the prosensory domain (Jacques et al., 2012). To determine whether the partial rescue of cochlear outgrowth observed in *Wls*^*cKO*^; *Gsk3*β^*cKO*^ mutants was due to activation of canonical Wnt signaling, we induced the expression of a stabilized β-catenin mutant in the cochlear epithelium by crossing an exon3-floxed β-catenin (*Ctnnb1^*flox(ex*3)^*) allele into *Wls*^*cKO*^ mutants. Deletion of exon3 driven by *Emx2*^*Cre*^ generated a mutant form of β-catenin refractory to inhibitory phosphorylation by Gsk3. Surprisingly, expression of stabilized β-catenin by itself resulted in a shortened cochlea, and cochlear outgrowth was severely stunted in the *Wls^cKO^*; *Ctnnb1*^Δ^ ^*ex*3/+^ compound mutants (Supplementary Figures 3C,D), precluding dissection and assessment of the OC. These results suggest that Gsk3β regulation of cochlear outgrowth is not mediated by stabilization of β-catenin.

### Effects of Rac1 Activation on Hair Bundle Defects of *Wls*^*cKO*^ Mutants

**FIGURE 4:**
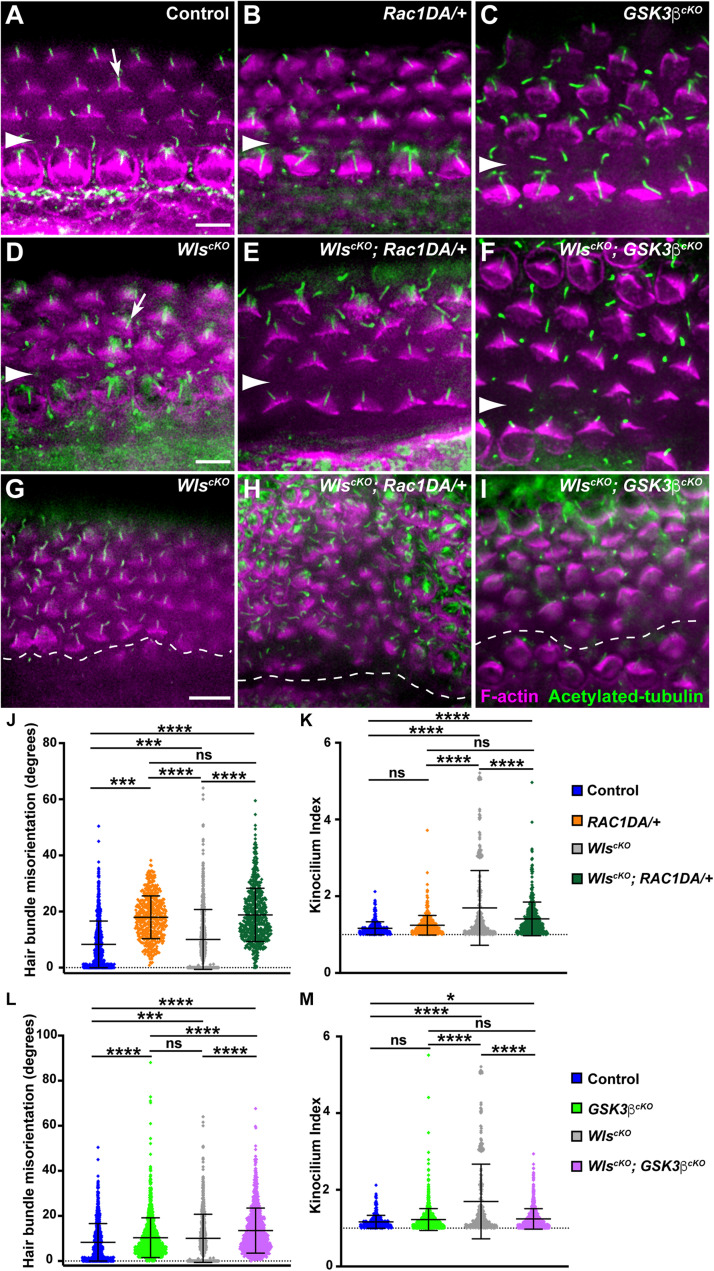
Partial rescue of hair bundle defects of *Wls*-deficient mutants by Rac1 activation and Gsk3β inactivation. **(A–I)** Flat-mounted E18.5 OC stained for acetylated tubulin (green) and F-actin (magenta). **(A–F)** Basal or mid-basal regions of wild-type control **(A)**, *Rac1DA/*+ **(B)**, *Gsk3*β^*cKO*^ **(C)**, *Wls*^*cKO*^ **(D)**, *Wls^cKO^*; *Rac1DA/*+ **(E)**, and *Wls*^*cKO*^; *Gsk3*β^*cKO*^ OC **(F)**. Arrows in panels **(A,D)** indicate normal kinocilium position at the hair bundle vertex and an off-center kinocilium, respectively. **(G–I)** Apical regions of *Wls*^*cKO*^ **(G)**, *Wls*^*cKO*^; *Rac1DA/*+ **(H)**, and *Wls*^*cKO*^; *Gsk3*β^*cKO*^ **(I)** OC. Arrowheads and the dashed line indicate the inner pillar cell row. Lateral is up. Scale bars: 6 μm. **(J–M)** Quantifications of hair bundle orientation **(J,L)** and kinocilium positioning **(K,M)**. Color keys for genotypes are indicated on the right. Ns, not significant.

Similar iPCP defects were observed in *Wls*^*cKO*^ and *Rac1*-deficient cochleae, including misoriented and misshapen hair bundles with an off-center kinocilium (Grimsley-Myers et al., 2009; Landin Malt et al., 2020), consistent with Rac1 being a downstream effector of Wnt-regulated hair bundle polarity. To test this, we examined hair bundle orientation and kinocilium positioning in *Wls^cKO^*; *Rac1DA/*+ cochleae at E18.5. Compared with the wild-type control, *Rac1DA/*+ had mild but significant hair bundle misorientation (Figures 4A,B,J and Supplementary Figure 4), consistent with the crucial role of localized Rac1 activity in hair bundle orientation (Grimsley-Myers et al., 2009). Interestingly, hair bundle misorientation in *Wls*^*cKO*^; *Rac1DA/*+ cochleae was more severe than the *Wls*^*cKO*^ mutants (Figures 4E,H,J and Supplementary Figure 4), particularly toward the cochlear apex where many supernumerary, disorganized OHC rows were present (Figures 4G,H).

We next assessed the effect of Rac1 activation on kinocilium positioning within the hair bundle by measuring the kinocilium index (Landin Malt et al., 2020). In the wild type at E18.5, the kinocilium is found at the vertex of the V-shaped hair bundle, with a mean kinocilium index (KI) of 1.16, whereas many hair bundles in *Wls*^*cKO*^ cochleae had an off-center kinocilium, as shown previously (Figures 4A,D, arrows). Rac1 activation by itself had negligible effect on the KI (mean = 1.24; Figures 4B,K). In the *Wls*^*cKO*^; *Rac1DA/*+ cochleae, kinocilium positioning was partially but significantly rescued compared with *Wls*^*cKO*^ mutants (Figures 4E,K). Thus, partial rescue of kinocilium positioning but not hair bundle orientation defects of *Wls*^*cKO*^ mutants by Rac1 activation supports the proposed role of Rac1 as a downstream effector of Wnt-mediated hair bundle polarity.

### The Role of Gsk3β in Hair Bundle Orientation and Kinocilium Positioning

To determine the role of Gsk3β in hair bundle morphogenesis, we first analyzed hair bundle orientation and kinocilium positioning in *Gsk3*β^*cKO*^ cochleae at E18.5. Interestingly, *Gsk3*β^*cKO*^ mutants had mild but significant hair bundle misorientation, indicating a requirement of Gsk3β for normal hair bundle orientation (Figures 4C,L and Supplementary Figure 4). On the other hand, the normal V-shape of the hair bundle and kinocilium positioning at the hair bundle vertex were largely intact in *Gsk3*β^*cKO*^ cochleae (Figures 4C,M).

Next, we assessed the effect of Gsk3β inactivation on hair bundle defects in *Wls*^*cKO*^ mutants. Interestingly, in *Wls^cKO^*; *Gsk3*β^*cKO*^ OC at E18.5, hair bundle misorientation was worse than in either single mutant (Figures 4F,I,L and Supplementary Figure 4). However, kinocilium positioning at the vertex of the hair bundle was significantly rescued compared with *Wls*^*cKO*^ mutants (Figures 4F,I,M). Taken together, these results indicate that a normal level of Gsk3β signaling is required for hair bundle orientation and that Wnts control hair bundle morphogenesis in part through inhibition of Gsk3β.

### Rac1 Activation and Gsk3β Inactivation Partially Restored Fzd6 and Dvl2 Junctional Localization in the Absence of Secreted Wnt Ligands

Our results so far suggest that both Rac1 and Gsk3β are downstream effectors of non-canonical Wnt signaling crucial for HC PCP. To further elucidate their roles in PCP establishment in the OC, we sought to determine whether Rac1 and Gsk3β also play a role in Wnt-dependent asymmetric localization of core PCP proteins.

**FIGURE 5:**
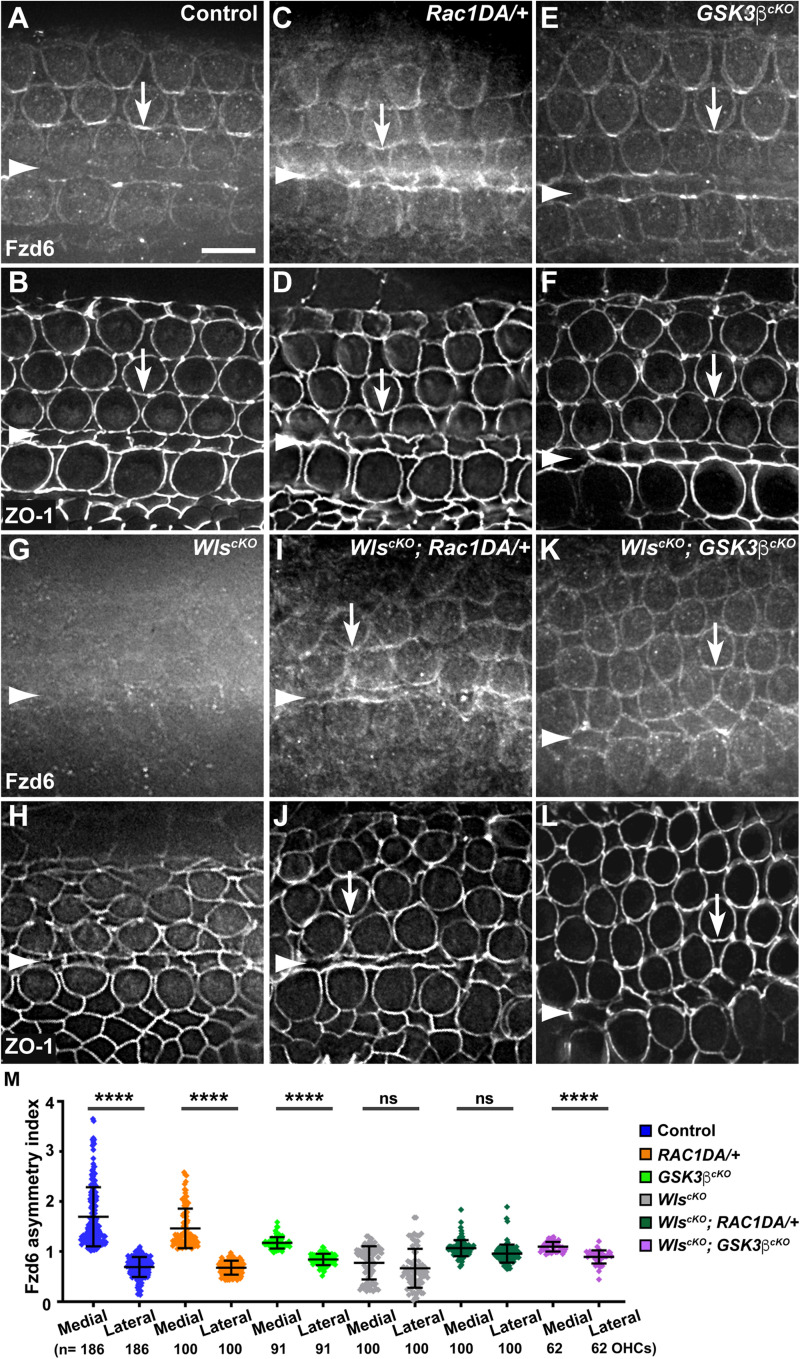
Rac1 activation and Gsk3β inactivation recovered Fzd6 junctional localization but not planar asymmetry in Wls-deficient OC. **(A–L)** Flat-mounted E18.5 wild-type control **(A,B)**, *Rac1DA/*+ **(C,D)**, *Gsk3*β^*cKO*^ **(E,F)**, *Wls*^*cKO*^ **(G,H)**, *Wls*^*cKO*^; *Rac1DA/*+ **(I,J)**, and *Wls*^*cKO*^; *Gsk3*β^*cKO*^ **(K,L)** OC stained for Fzd6 and ZO-1 as indicated. Arrows indicate Fzd6 crescents along the medial borders of OHCs. Arrowheads indicate the inner pillar cell row. Lateral is up. Scale bar: 6 μm. **(M)** Quantifications of Fzd6 staining along the medial and lateral junctions of OHCs. Numbers of OHCs scored are indicated on the bottom. Color keys for genotypes are indicated on the right. Ns, not significant.

We and others previously uncovered a requirement of secreted Wnt ligands in asymmetric junctional localization of a subset of core PCP proteins (Landin Malt et al., 2020; Najarro et al., 2020). Specifically, Fzd6 is normally enriched along the medial border of HCs (Figures 5A,B, arrows, 7A), and this localization was abolished in the *Wls*^*cKO*^ cochleae (Figures 5G,H, 7D). Similar to the control, we found that Fzd6 was enriched along medial HC junctions in both the *Rac1DA/*+ and *Gsk3*β^*cKO*^ cochleae (Figures 5C–F, 7B,C). Interestingly, junctional Fzd6 localization was significantly recovered in both *Wls*^*cKO*^; *Rac1DA/*+ and *Wls*^*cKO*^; *Gsk3*β^*cKO*^ cochleae; however, Fzd6 planar asymmetry along the medial-lateral axis was not restored in *Wls*^*cKO*^; *Rac1DA/*+ and only partially restored in *Wls*^*cKO*^; *Gsk3*β^*cKO*^ cochleae (Figures 5I–M, 7E,F).

**FIGURE 6:**
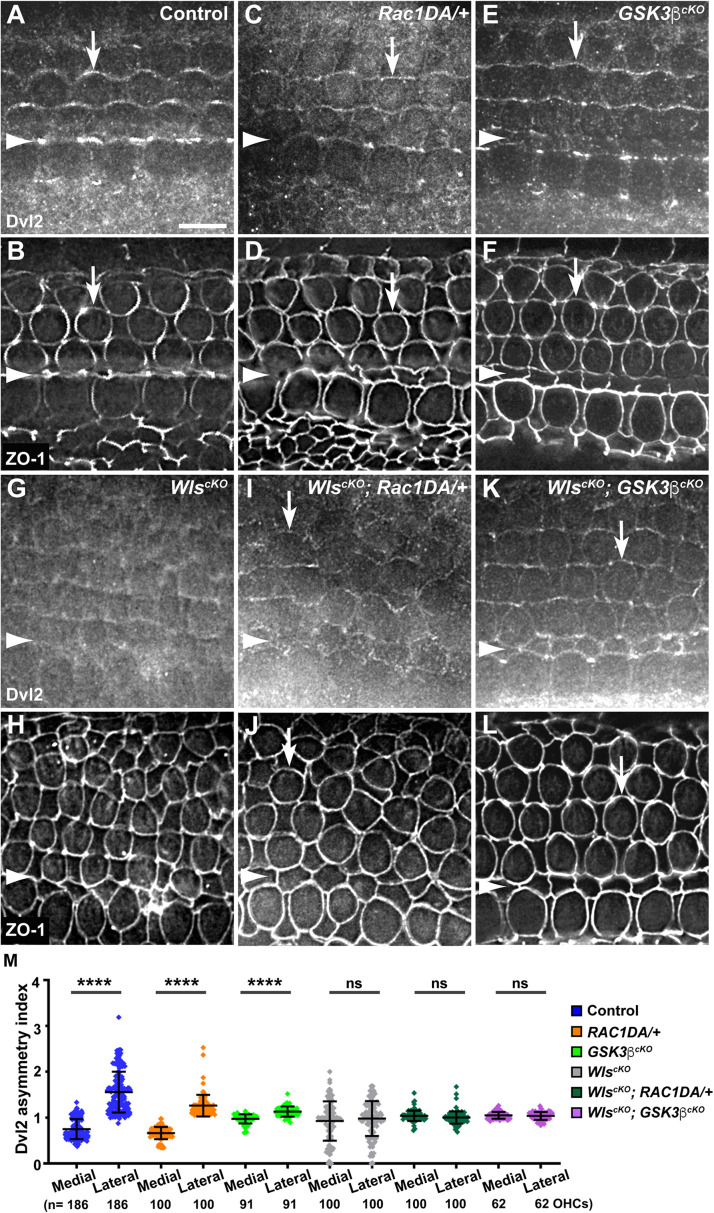
Effects of Rac1 activation and Gsk3β inactivation on Dvl2 localization in Wls-deficient OC. **(A–L)** Flat-mounted E18.5 wild-type control **(A,B)**, *Rac1DA/*+ **(C,D)**, *Gsk3*β^*cKO*^ **(E,F)**, *Wls*^*cKO*^ **(G,H)**, *Wls*^*cKO*^; *Rac1DA/*+ **(I,J)**, and *Wls*^*cKO*^; *Gsk3*β^*cKO*^ **(K,L)** OC stained for Dvl2 and ZO-1 as indicated. Arrows indicate Dvl2 crescents along the lateral borders of OHCs. Arrowheads indicate the inner pillar cell row. Lateral is up. Scale bar: 6 μm. **(M)** Quantifications of Dvl2 staining along the medial and lateral junctions of OHCs. Numbers of OHCs scored are indicated on the bottom. Color keys for genotypes are indicated on the right. Ns, not significant.

**FIGURE 7:**
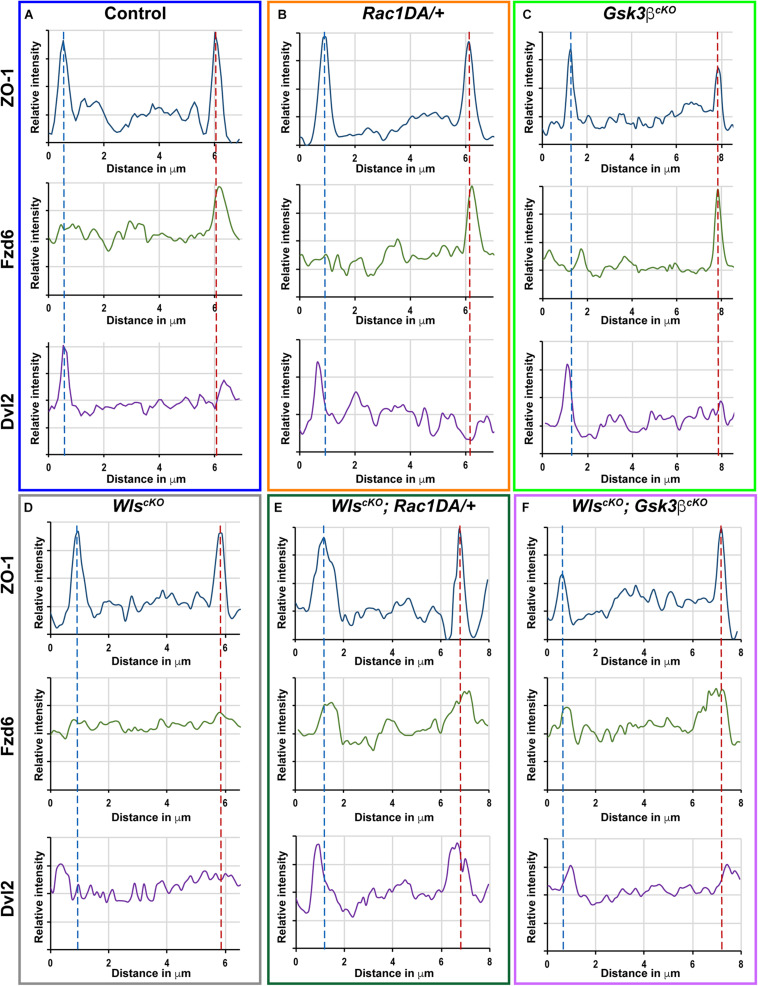
Linescan analysis of junctional localization of Fzd6 and Dvl2 in outer hair cells. **(A–F)** Representative linescans of individual OHCs from E18.5 wild-type control **(A)**, *Rac1DA/*+ **(B)**, *Gsk3*β^*cKO*^ **(C)**, *Wls*^*cKO*^ **(D)**, *Wls^cKO^*; *Rac1DA/*+ **(E)**, and *Wls*^*cKO*^; *Gsk3*β^*cKO*^ **(F)** OC stained for ZO-1, Fzd6, and Dvl2. For each genotype, a line was drawn parallel to the medial–lateral axis bisecting the OHC. Intensity profiles of each image channel were aligned along the distance axis. The lateral and medial junctions of the OHC were identified by peaks of ZO-1 staining and indicated by the blue and red dashed lines, respectively. Junctional Fzd6 and Dvl2 staining was defined by peaks in close proximity to the lateral or medial borders of the OHC.

Another Wnt-dependent core PCP protein, Dvl2, is normally enriched along the lateral border of HCs (Figures 6A,B, arrows, 7A) and lost its junctional localization in the *Wls*^*cKO*^ cochleae (Figures 6G,H, 7D). In both the *Rac1DA/*+ and *Gsk3*β^*cKO*^ cochleae, enrichment of Dvl2 on the lateral HC junctions was largely intact (Figures 6C–F, arrows, 7B,C). In *Wls^cKO^*; *Rac1DA/*+ and *Wls*^*cKO*^; *Gsk3*β^*cKO*^ OC, junctional Dvl2 localization was partially recovered (Figures 6I–L, arrows, 7E,F). However, Dvl2 planar asymmetry was not restored in either compound mutant (Figure 6M). Together, these results indicate that both Rac1 and Gsk3β are involved in Wnt-mediated junctional localization of a subset of core PCP proteins; however, neither Rac1 activation nor Gsk3β inactivation was sufficient for generating planar asymmetry of core PCP proteins.

## Discussion

### The Non-canonical Wnt Pathway Signals Through Multiple Effectors to Control Different Aspects of Cochlear Morphogenesis

**FIGURE 8:**
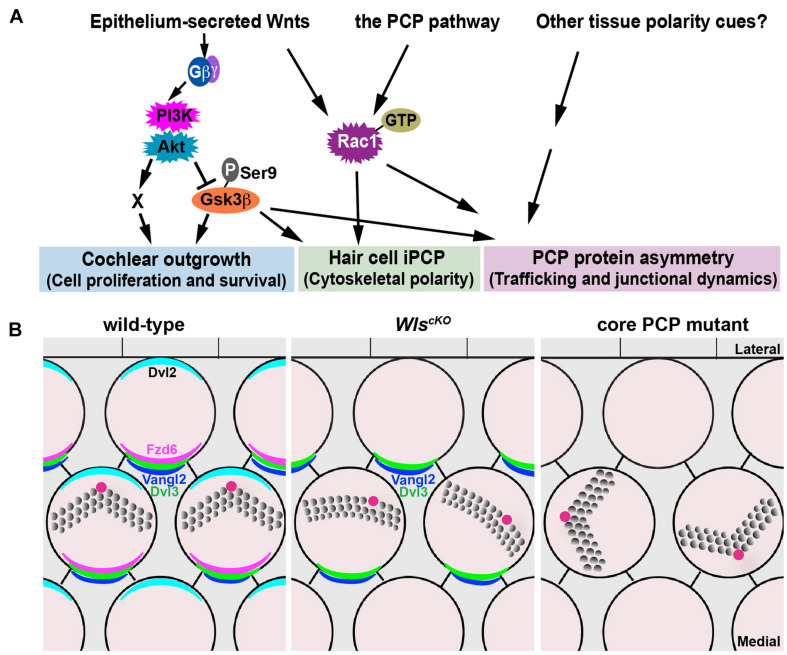
Non-canonical Wnt and PCP pathways act in concert to regulate cochlear morphogenesis. **(A)** A working model for the concerted actions of the non-canonical Wnt and PCP pathways in regulating different aspects of cochlear morphogenesis. **(B)** Schematic diagrams comparing PCP defects in the *Wls*^*cKO*^ and core PCP mutant OC. Kinocilium (the red dot) positioning at the hair bundle vertex was disrupted in *Wls*^*cKO*^ but not core PCP mutants.

In this study, we have further delineated the non-canonical, Wnt/G-protein/PI3K pathway for cochlear outgrowth and establishment of iPCP and PCP in the cochlea (Figure 8A). Our genetic rescue experiments have provided strong evidence that PI3K, Gsk3β, and Rac1 are all downstream effectors of non-canonical Wnt signaling in the cochlea. The extent to which the cochlear defects of *Wls*^*cKO*^ mutants were rescued varied among the effectors. Thus, these effectors likely act in parallel and have non-overlapping functions to mediate non-canonical Wnt signaling in the cochlea.

Cochlear elongation is regulated by multiple developmental signals from the epithelium and surrounding mesenchyme and spiral ganglion (Bok et al., 2013; Huh et al., 2015), suggesting integration of multiple molecular pathways. Within the epithelium, while PCP signaling is thought to mediate convergent extension of the OC (Mao et al., 2011; Montcouquiol and Kelley, 2019), the mechanisms underlying Wnt-mediated cochlear elongation remain incompletely understood. We showed that outgrowth of *Wls*^*cKO*^ cochleae was rescued fully by PI3K activation and partially by Gsk3β inactivation but not rescued by Rac1 activation or expression of stabilized β-catenin, suggesting that Wnt/G-protein/PI3K signaling engages Gsk3β and additional regulators (“X”; Figure 8A) to promote cell proliferation and/or cell survival during cochlear outgrowth.

### Epithelium-Secreted Wnts Act in Parallel to and Cross-Regulates the PCP Pathway in the Cochlea

Our findings suggest that Wnts secreted by the cochlear epithelium act in parallel and crosstalk with the PCP pathway in the mammalian cochlea. Of note, *Wls*^*cKO*^ and core PCP mutants have distinct hair bundle phenotypes: hair bundle misorientation in *Wls*^*cKO*^ cochleae was milder than core PCP mutants; moreover, *Wls*^*cKO*^ but not PCP mutants were defective in kinocilium positioning at the hair bundle vertex (Figure 8B). Epithelium-secreted Wnt ligands likely act in concert with additional tissue polarity cues, including non-epithelial Wnts, to specify the PCP vector and align HC orientation (Figure 8A). Epithelium-secreted Wnt5a, a prototype non-canonical Wnt, is dispensable for cochlear PCP (Najarro et al., 2020), suggesting involvement of other Wnt ligands. In the future, identification of the relevant Wnt ligands in the cochlea will help determine permissive versus instructive roles of Wnt signaling in HC PCP. Importantly, we have uncovered a non-canonical Wnt pathway that signals through PI3K, Rac1, and Gsk3β to promote junctional localization of a subset of core PCP proteins, including Fzd6 and Dvl2, thereby cross-regulating the PCP pathway. This is in stark contrast to the *Drosophila* PCP pathway, which operates independently of Wnt ligands (Bartscherer et al., 2006; Chen et al., 2008; Ewen-Campen et al., 2020; Yu et al., 2020). Wnt signaling may regulate the trafficking of PCP proteins or HC–SC junctional dynamics, which in turn influences asymmetric PCP protein localization (see below).

### Rac1 Integrates Multiple Developmental Signals During Hair Cell Planar Polarization

Although Rac1 is activated by non-canonical Wnt signaling in cultured cells, it remains to be determined whether Wnt signaling stimulates Rac1 activity in the cochlea. At present, we have been unable to address this question, as our attempts to evaluate the localization and levels of active Rac1 by immunostaining or Western blot using a commercially sourced Rac1-GTP-specific antibody were unsuccessful. Thus, more sensitive and specific tools are needed to detect active Rac1 *in vivo*. PCP defects caused by modest overexpression of Rac1-G12V expression were mild, likely due to the presence of wild-type Rac proteins undergoing the normal GTPase cycle. In the *Wls^cKO^*; *Rac1DA/*+ OC, Fzd6 and Dvl2 junctional localization was partially rescued, consistent with Rac1 being a downstream effector of non-canonical Wnt signaling. Rac1 may promote Fzd6 and Dvl2 junctional localization through regulation of junctional and cytoskeletal dynamics (de Curtis and Meldolesi, 2012). In previous studies, we have shown that the activity of p21-activated kinases (PAKs), which are downstream effectors of both Rac1 and Cdc42, are regulated in the OC by multiple mechanisms, including intercellular PCP signaling, plus- and minus-end-directed microtubule motors and the cell polarity protein Par3 (Grimsley-Myers et al., 2009; Sipe and Lu, 2011; Sipe et al., 2013; Landin Malt et al., 2019). Therefore, multiple signaling pathways, including the non-canonical Wnt pathway, likely converge on Rac1 to tightly control its activity in space and time during HC planar polarization.

### Gsk3β Inhibition Is a Key Step of the Non-canonical Wnt Pathway in the Cochlea

We show, for the first time, that Wnts secreted by the cochlear epithelium promote inhibitory Ser9 phosphorylation of Gsk3β *in vivo*. This is different from the mode of Gsk3β inhibition by canonical Wnt signaling, which is thought to occur through sequestration of Gsk3β and dissociation of the disruption complex (Metcalfe and Bienz, 2011; Beurel et al., 2015). Previous studies using pharmacological inhibitors have shown that Gsk3 signaling regulates OC progenitor cell proliferation and fate decision (Jacques et al., 2012; Ellis et al., 2019). Our genetic analyses further reveal multi-faceted roles of Gsk3β in PCP and iPCP regulation *in vivo*. First, Gsk3β is required for uniform hair bundle orientation. Second, Gsk3β inhibition is crucial for Wnt-dependent kinocilium positioning and junctional localization of Fzd6 and Dvl2. Interestingly, both too little (in *Gsk3*β^*cKO*^ mutants) and too much Gsk3β activity (in *Wls*^*cKO*^ mutants) led to hair bundle orientation defects, suggesting that levels of Gsk3β need to be precisely controlled to achieve uniform HC orientation. In the future, it would be interesting to assess earlier roles of Gsk3β in cochlear patterning *in vivo* by deleting Gsk3β in the otocyst.

Gsk3β is a promiscuous kinase with numerous known substrates. The crucial Gsk3β targets that mediate kinocilium positioning and PCP protein localization remain to be identified. Gsk3β has a well-established role in regulating neuronal cytoskeletal dynamics through phosphorylation of microtubule-associated proteins (MAPs) including collapsin response mediator proteins (CRMPs), APC, Tau, MAP1B, and doublecortin (Hur and Zhou, 2010; Morgan-Smith et al., 2014). In the OC, microtubules and microtubule-based motors have been implicated in hair bundle orientation and kinocilium positioning (Sipe and Lu, 2011; Ezan et al., 2013; Sipe et al., 2013). In addition, cytoskeletal molecules disrupted in Usher syndrome and ciliopathies also play a role in hair bundle polarity (Lefevre et al., 2008; Jagger et al., 2011). Thus, HC microtubule and other cytoskeletal regulators are potential targets of Gsk3β during hair bundle morphogenesis.

In other systems, microtubules are also involved in polarized trafficking of core PCP proteins (Vladar et al., 2012; Matis et al., 2014). However, asymmetric PCP protein localization was normal in several mutants affecting HC microtubule organization or transport (Sipe and Lu, 2011; Kirjavainen et al., 2015; Siletti et al., 2017), suggesting alternative mechanisms by which Gsk3β regulates PCP protein localization/trafficking. Interestingly, Gsk3β has been shown to regulate endocytosis/recycling of membrane cargos in different cell types (Roberts et al., 2004; Clayton et al., 2010; Reis et al., 2015; Ferreira et al., 2020). Future investigations will shed light on the mechanisms by which Gsk3β influences HC polarity and core PCP protein trafficking in the OC.

## Materials and Methods

### Mice

Animal care and use was performed in compliance with the NIH guidelines and the Institutional Animal Care and Use Committee at the University of Virginia. *Wls*^*flox*^ (Fu et al., 2011), *Gsk3*β^*flox*^ (Patel et al., 2008), and *R26-LSL-Rac1DA* (Srinivasan et al., 2009) mice were obtained from the Jackson Laboratories (Stock #012888, #029592, and #012361, respectively). *Ctnnb1^*flox(ex*3)^* and *Emx2*^*Cre*^ mice have been described (Harada et al., 1999; Ono et al., 2014). All mice were maintained on a mixed genetic background. To generate *Wls* conditional and compound mutants, *Wls^*flox/+*^*; Emx2^*Cre/+*^ males were mated with *Wls*^*flox/flox*^, *Wls^*flox/flox*^*; *R26-LSL-Rac1DA/*+, or *Wls^*flox/flox*^*; *Ctnnb1*^*flox(ex*3)/+^ females, and *Wls^*flox/+*^*; *Gsk3*β^*flox/+*^; *Emx2*^*Cre/+*^ males with *Wls^*flox/flox*^*; *Gsk3*β^*flox/flox*^ females. For timed pregnancies, the morning of the plug was designated as embryonic day 0.5 (E0.5), and the day of birth postnatal day 0 (P0).

### Immunohistochemistry

Mouse skulls were dissected and fixed in 4% paraformaldehyde (PFA) for 45 min at room temperature (RT) or in 10% TCA for 1 h on ice, then washed three times in PBS. Dissected cochleae were blocked in PBS containing 0.1% Triton X-100, 5% heat-inactivated horse serum, and 0.02% NaN3 for 1 h at RT, then incubated with primary antibodies for 16–32 h at 4°C. After three washes in PBS/0.1% Triton X-100, samples were incubated with secondary antibodies and phalloidin for 2 h at RT, washed twice in PBS 0.1% Triton X-100, post-fixed for 15 min at RT in 4% paraformaldehyde and then washed two more times. Stained samples are flat mounted in Mowiol with 5% N-propyl gallate. The table above lists the antibodies used.

**Table:** 

Antibody	Concentration	Vendor	Catalog No.
Alexa-conjugated phalloidin	1:200	Thermo Fisher Scientific	A12379, A12380, A12381, A22287
Alexa-conjugated secondary antibodies	1:500	Thermo Fisher Scientific	A11036, A11029, A11057, A11077
Anti-acetylated tubulin	1:1,000	Sigma-Aldrich	T6793
Anti-Dvl2	1:100 (TCA)	Proteintech	12037-1-AP
Anti-Fzd6	1:100 (TCA)	R & D Systems	AF1526-SP
Anti-S9-Gsk3β (D85E12)	1:100	Cell Signaling	5558
Anti-Gsk3β (D5C5Z)	1:100 (TCA)	Cell Signaling	12456
Anti-myosin VI	1:500	PROTEUS	25-6791
Anti-ZO-1	1:100 (TCA)	DSHB	R26.4C-c

### Microscopy and Image Analysis

Control and mutant samples were imaged under identical conditions. For hair bundle and PCP protein localization, images were collected using a Deltavision deconvolution microscope with a 60×/1.35 NA oil-immersion objective controlled by SoftWoRx software (Applied Precision). Whole-mount cochlear ducts were imaged using a Leica MZ16F stereomicroscope. Images were processed using Fiji (National Institutes of Health) and Photoshop (Adobe).

### Quantification of Hair Bundle Phenotypes

Hair bundle orientation and kinocilium position within the hair bundle along the entire cochlear length were quantified as previously described (Landin Malt et al., 2020). In brief, the hair bundle is labeled by phalloidin staining and the kinocilium by anti-acetylated tubulin staining. A hair bundle with its vertex pointing to the lateral or medial edge of the cochlear duct has a misorientation of 0° and 180°, respectively. The kinocilium index is the length ratio of the long and short hair bundle “halves” as bisected by the kinocilium.

### Quantification of Protein Localization

Fzd6 and Dvl2 immunostaining along OHC-Deiters cell junctions from the basal to mid-apical regions was quantified using Fiji, as previously described (Landin Malt et al., 2020). The cochlear apex, where HC–SC junctions were more irregular/less mature, was excluded. In brief, single optic sections with the strongest junctional staining intensity were chosen for each imaging channel. In general, lateral Dvl2 crescents are localized at the level of the tight junction, while medial Fzd6 crescents are localized at a level about 1 μm below the tight junction. Cell junctions were identified using ZO-1 staining. A 30 × 10−, 20 × 10−, and 30 × 5-pixel region of interest (ROI) centered around the medial, lateral, and orthogonal OHC junctions, respectively, was then selected. Following background subtraction, mean fluorescence intensity of Fzd6 or Dvl2 staining for the medial or lateral ROI was normalized to that of the orthogonal ROI of the same OHC and plotted as the asymmetry index.

Line scan analysis was performed using Fiji and Excel to demonstrate the localization of Fzd6 and Dvl2 staining relative to cell junctions. Specifically, a diametral line was drawn intersecting the lateral and medial OHC junctions as marked by ZO-1 immunostaining. Following background subtraction, fluorescence intensity of each imaging channel was plotted in the lateral to medial direction of the line.

## Statistics

Statistical analysis of at least three cochleae from three different litters was performed using GraphPad Prism. Data were analyzed using one-way analysis of variance (ANOVA) followed by a *post hoc* Tukey’s test. *p*-Values for statistical significance are defined as follows: ^∗^*p* ≤ 0.0332; ^∗∗^*p* ≤ 0.0021; ^∗∗∗^*p* ≤ 0.0002, and ^****^p ≤ 0.0001. Data were presented as mean ± standard deviation.

## Data Availability Statement

The original contributions presented in the study are included in the article/Supplementary Material, further inquiries can be directed to the corresponding author.

## Ethics Statement

The animal study was reviewed and approved by the Animal Care and Use Committee at the University of Virginia.

## Author Contributions

ALM and XL designed the research, performed the experiments, analyzed the data, and wrote the manuscript. SC performed the experiments and analyzed the data. DH, AL, CS, MS, MH, and CC analyzed the data. All authors contributed to the article and approved the submitted version.

## Conflict of Interest

The authors declare that the research was conducted in the absence of any commercial or financial relationships that could be construed as a potential conflict of interest.
