# Supplementary material for: Non-Canonical Wnt Signaling Regulates Cochlear Outgrowth and Planar Cell Polarity via Gsk3β Inhibition
Source: Front Cell Dev Biol. 2021 Apr 16;9:649830. doi: 10.3389/fcell.2021.649830 (PMC8086559; doi:10.3389/fcell.2021.649830)
Supplement: Supplementary file 1 [file Data_Sheet_1.docx]

**­­
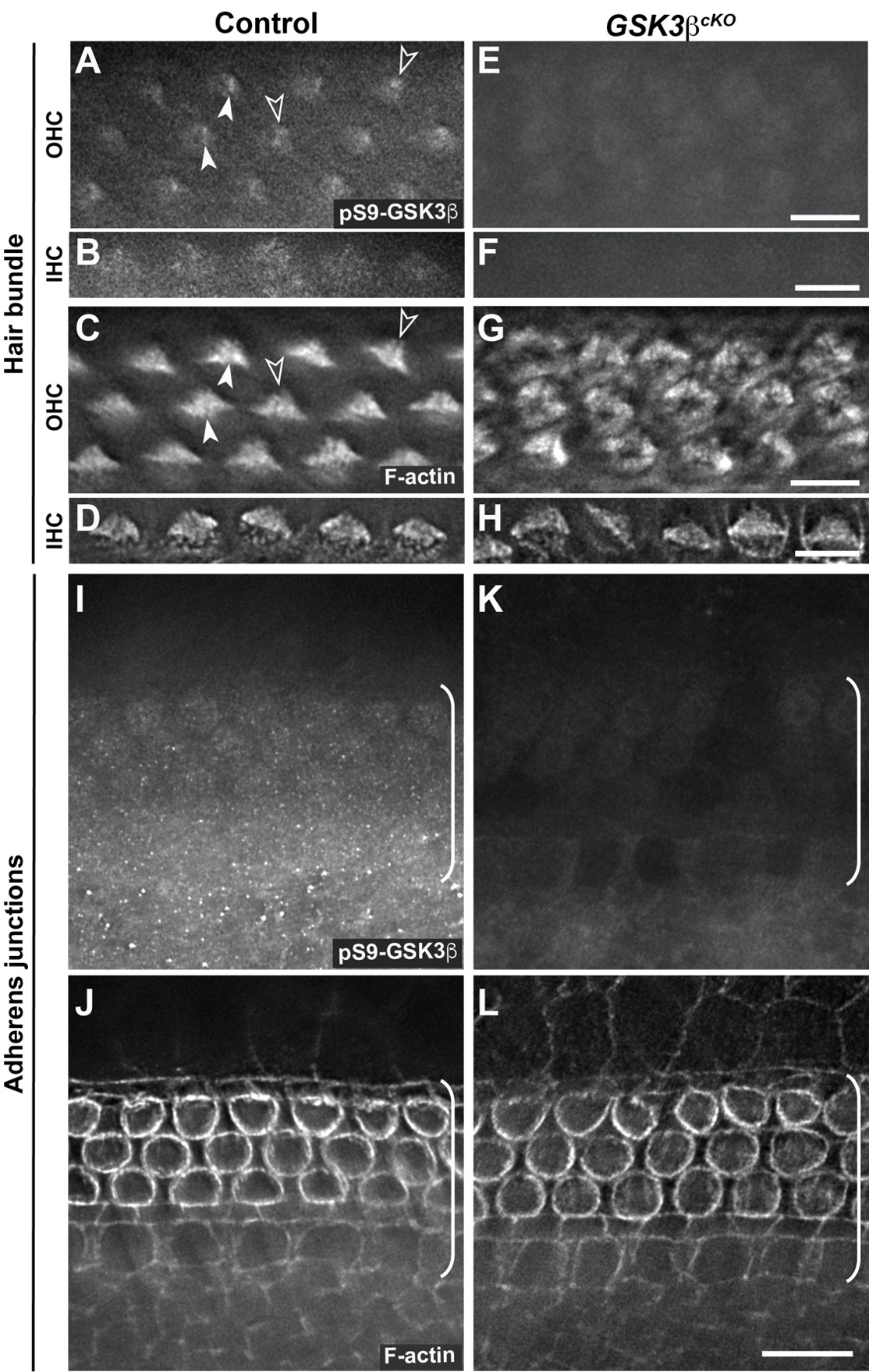
**

**Supplementary Figure 1. Specific staining patterns of pS9-Gsk3β in the cochlea.**

(A-H) pS9-Gsk3β and phalloidin staining at the level of hair bundle in control (A-D) and *Gsk3β^cKO^* (E-H) OC at E18.5. Open arrowheads indicate the pericentriolar region. Arrowheads indicate the kinocilium. (I-L) pS9-Gsk3β and phalloidin staining at the level of adherens junctions in control (I, J) and *Gsk3β^cKO^* (K, L) OC and surrounding regions. Brackets indicate the OC. Lateral is up. Scale bars: A-H, 6 µm; I-L, 10 µm.

**
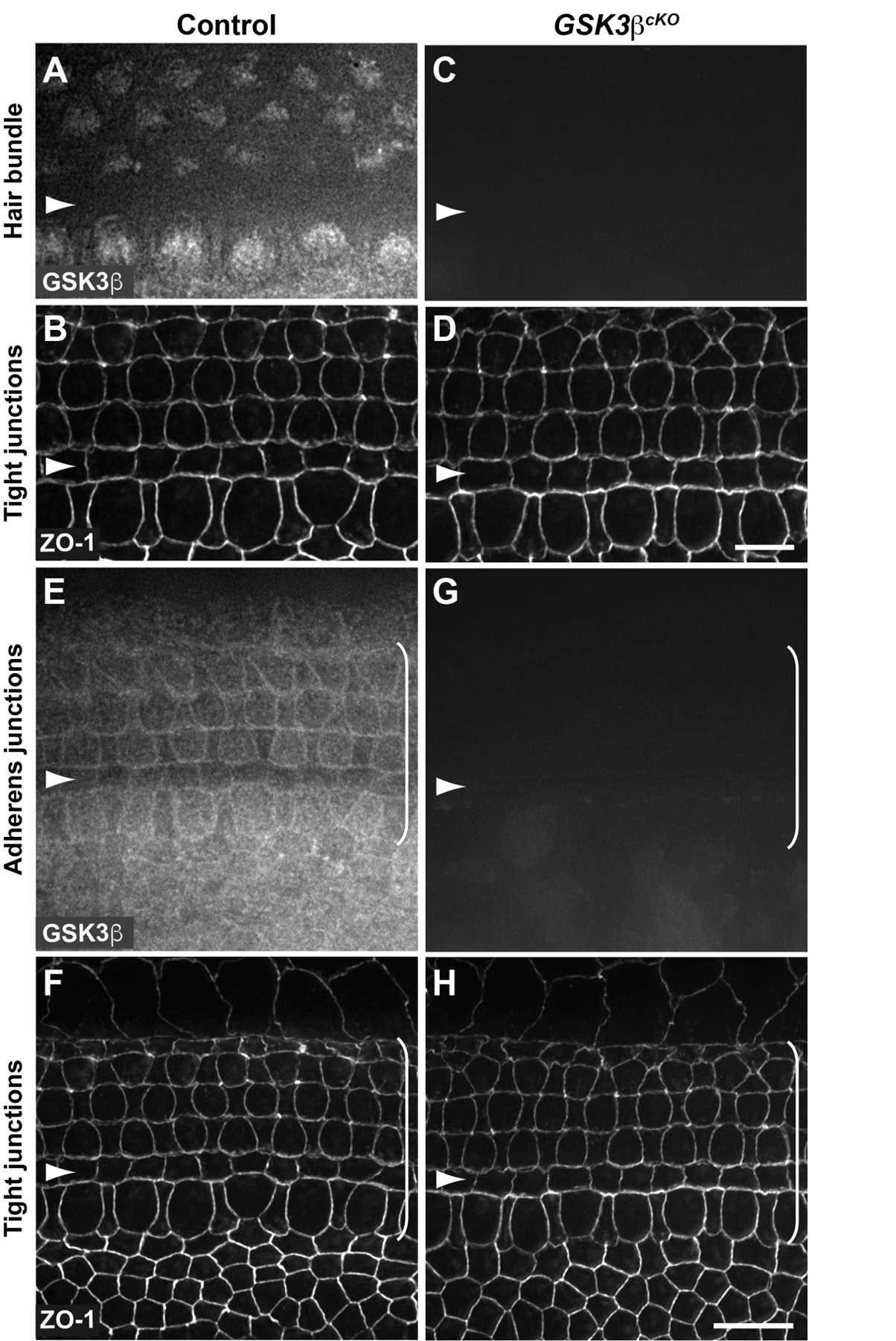
**

**Supplementary Figure 2. Specific staining patterns of Gsk3β in the cochlea.**

(A-D) Gsk3β staining at the level of hair bundle and ZO-1 staining at the level of tight junctions in control (A, B) and *Gsk3β^cKO^* (C, D) OC at E18.5. (E-H) Gsk3β staining at the level of adherens junctions and ZO-1 staining at the level of tight junctions in control (E, F) and *Gsk3β^cKO^* (G, H) OC and surrounding regions. Brackets indicated the OC. Lateral is up. Scale bars: A-D, 6 µm; E-H, 10 µm.


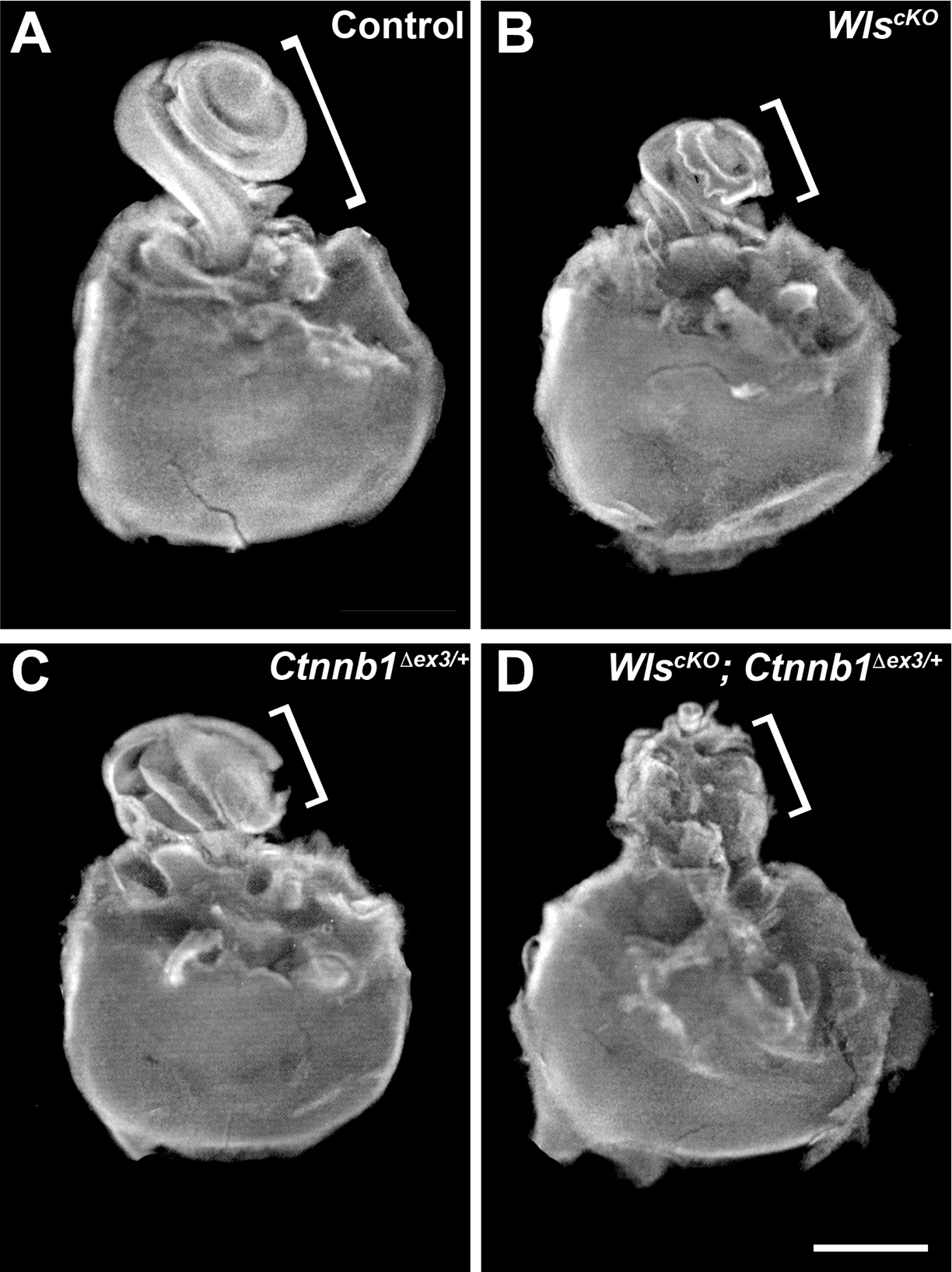


**Supplementary Figure 3. Cochlear outgrowth in *Wls^cKO^; Ctnnb1^Δex3/+^* mutants was severely stunted.**

(A-D) E18.5 control (A), *Wls^cKO^* (B), *Ctnnb1^Δex3/+^* (C) and *Wls^cKO^; Ctnnb1^Δex3/+^* (D) otic capsules partially dissected to expose the cochlear duct. Brackets indicate the cochlea. Scale bar: 1 mm.

**
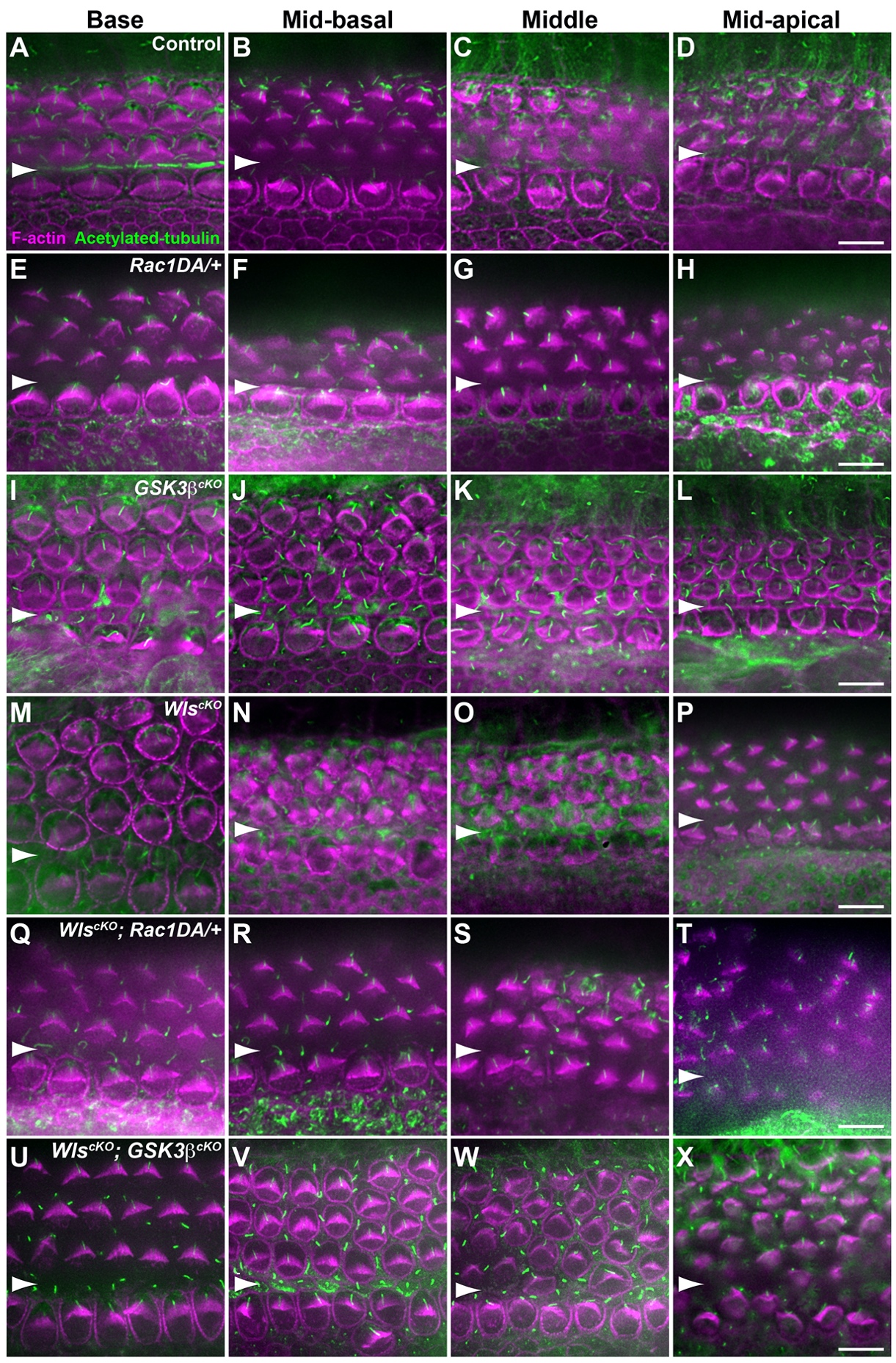
**

**Supplementary Figure 4. Hair bundle staining along the longitudinal axis of the cochlea.**

(A-X) Flat-mounted OC stained for acetylated tubulin (green) and F-actin (magenta). The positions along the longitudinal axis of the cochlea are indicated on top. The genotypes are: (A-D) Control, (E-H) *Rac1DA/+*, (I-L) *Gsk3b^cKO^*, (M-P) *Wls^cKO^*, (Q-T) *Wls^cKO^; Rac1DA/+*, and (U-X) *Wls^cKO^*; *Gsk3b^cKO^*. Arrowheads indicate the inner pillar cell row. Lateral is up. Scale bars: 6 μm.
